# Request for organ donation without donor registration: a qualitative study of the perspectives of bereaved relatives

**DOI:** 10.1186/s12910-016-0120-6

**Published:** 2016-07-11

**Authors:** Jack de Groot, Maria van Hoek, Cornelia Hoedemaekers, Andries Hoitsma, Hans Schilderman, Wim Smeets, Myrra Vernooij-Dassen, Evert van Leeuwen

**Affiliations:** Radboud Institute for Health Sciences, Radboud University Medical Center, DGVP 20, PO Box 9101, 6500 HB Nijmegen, The Netherlands; Department of Spiritual and Pastoral Care, Radboud University Medical Center, Nijmegen, The Netherlands; Department of Intensive Care Medicine, Radboud University Medical Centre, Nijmegen, The Netherlands; Department of Nephrology, Radboud University Medical Center, Nijmegen, The Netherlands; Faculty of Philosophy, Theology and Religious Studies, Radboud University Nijmegen, Nijmegen, The Netherlands; Kalorama Foundation, Nijmegen, The Netherlands

**Keywords:** Decision making by proxies, Donor registration, Ethics, Organ transplantation, Professional-family relations, Informational needs

## Abstract

**Background:**

In the Netherlands, consent from relatives is obligatory for post mortal donation. This study explored the perspectives of relatives regarding the request for consent for donation in cases without donor registration.

**Methods:**

A content analysis of narratives of 24 bereaved relatives (14 in-depth interviews and one letter) of unregistered, eligible, brain-dead donors was performed.

**Results:**

Relatives of unregistered, brain-dead patients usually refuse consent for donation, even if they harbour pro-donation attitudes themselves, or knew that the deceased favoured organ donation. Half of those who refused consent for donation mentioned afterwards that it could have been an option. The decision not to consent to donation is attributed to contextual factors, such as feeling overwhelmed by the notification of death immediately followed by the request; not being accustomed to speaking about death; inadequate support from other relatives or healthcare professionals, and lengthy procedures.

**Conclusion:**

Healthcare professionals could provide better support to relatives prior to donation requests, address their informational needs and adapt their message to individual circumstances. It is anticipated that the number of consenting families could be enlarged by examining the experience of decoupling and offering the possibility of consent for donation after circulatory death if families refuse consent for donation after brain-death.

**Electronic supplementary material:**

The online version of this article (doi:10.1186/s12910-016-0120-6) contains supplementary material, which is available to authorized users.

## Background

Registration of the organ donation preferences of the eligible donor on a driver’s license or in an official donor register has generally been recognised as the major reason why relatives consent to organ donation [1–5]. However, only 44 % of the adult population of the Netherlands has registered in the Dutch National Donor Register. Most of the eligible (brain-dead) organ donors are consequently not registered and their relatives often have to decide about donation without knowing the preference of the deceased. It is assumed that this situation easily leads to refusal of consent for donation [6]. These and other important facts related to transplantation in the Netherlands are summarised in Additional file 1.

In a previous article [7], it was ascertained that participants always follow the registered wish to donate. When, however, the preference for donation was unknown or informally communicated, families usually refused consent for donation. Additionally we found that situational factors have more influence on the decision to consent to donation than considerations regarding values and motives. This study focused on a unique subsample that contained only relatives of eligible donors without donor registration.

The aim was to obtain insight into the factors that influence the decision-making process of relatives of unregistered eligible, brain dead donors, and whether these factors are related to the outcome of the request. The relatives’ perspective includes experiences, evaluation of the circumstances of the request, accountability for the decision and the way that relatives give meaning to the motives that play a role in their decision. Although more than half of the Dutch population are in favour for organ donation [8], most families refuse consent for donation on behalf of their relatives, when there is no donor registration (see Additional file 1). The ultimate aim of the study was to determine key factors in the decision-making process from the perspective of families who must decide in absence of this donor registration.

## Methods

### Research design

A secondary analysis of data from a previous qualitative study [7] with registered and non-registered potential organ donors was conducted. To account for the differences between registered and non-registered potential donors, a new content analysis of the face-to-face, in-depth interviews with relatives of eligible, but not registered, organ donors was performed. The interviews were made with the use of a topic guide (Additional file 2). The topics were derived from the research questions, a review of literature [9] and the authors’ experience in the field of organ donation.

### Recruitment period and procedure

The secondary analysis includes 14 of the 22 cases of the previous study, in which the brain dead eligible donors were not registered in the Dutch National Donor Register.

All participants of the total study were proxies of potential donors from the Radboud university medical centre in Nijmegen and the Sint Elisabeth Hospital in Tilburg, both in the Netherlands, between 1^st^ October 2008 and 30^th^ September 2012. In this period, all relatives who were confronted with the donation request were asked to disclose their address. The Primary Researcher received the name of 52 main proxies, who gave consent to the treating physician or the transplant coordinator to disclose their addresses. At least 6 weeks after death of the relative, the proxy was invited by letter for an interview alone or with additional significant others, regarding their experiences with donation request. About half of the proxies reacted positively on this letter; however, some refused the interview because of the sensitive nature of the topic.

This secondary analysis examines the 14 cases in which the eligible donors were not registered in the Dutch National Donor Register. We interviewed 23 participants (six singles, seven pairs and one trio) and received a letter from a 24^th^ person. The eight cases in which the deceased was registered as organ donor were excluded from the secondary analysis. Prior to the recruitment process, permission from the Research Ethics Committees of the two hospitals was obtained.

### Data collection and measurement

All in-depth interviews were carried out by the Primary Researcher. Interviews took place approximately 3.5 months after the death of the relative and lasted between 43 and 90 min (mean = 65 min). All interviews were recorded with a voice recorder and transcribed into text format by an assistant. All transcripts were summarised on the topics of the interview (Additional file 2). This summary was approved by the participants in a telephone call that was made by the Primary Researcher. The Researcher also enquired in the same telephone call with participants, if aftercare was required, because of the possible emotional impact of the interview. None of the participants requested this support; some of them revealed that they were still receiving support from a psycho-social caregiver (psychologist, vicar, social worker).

### Analysis

**Fig. 1:**
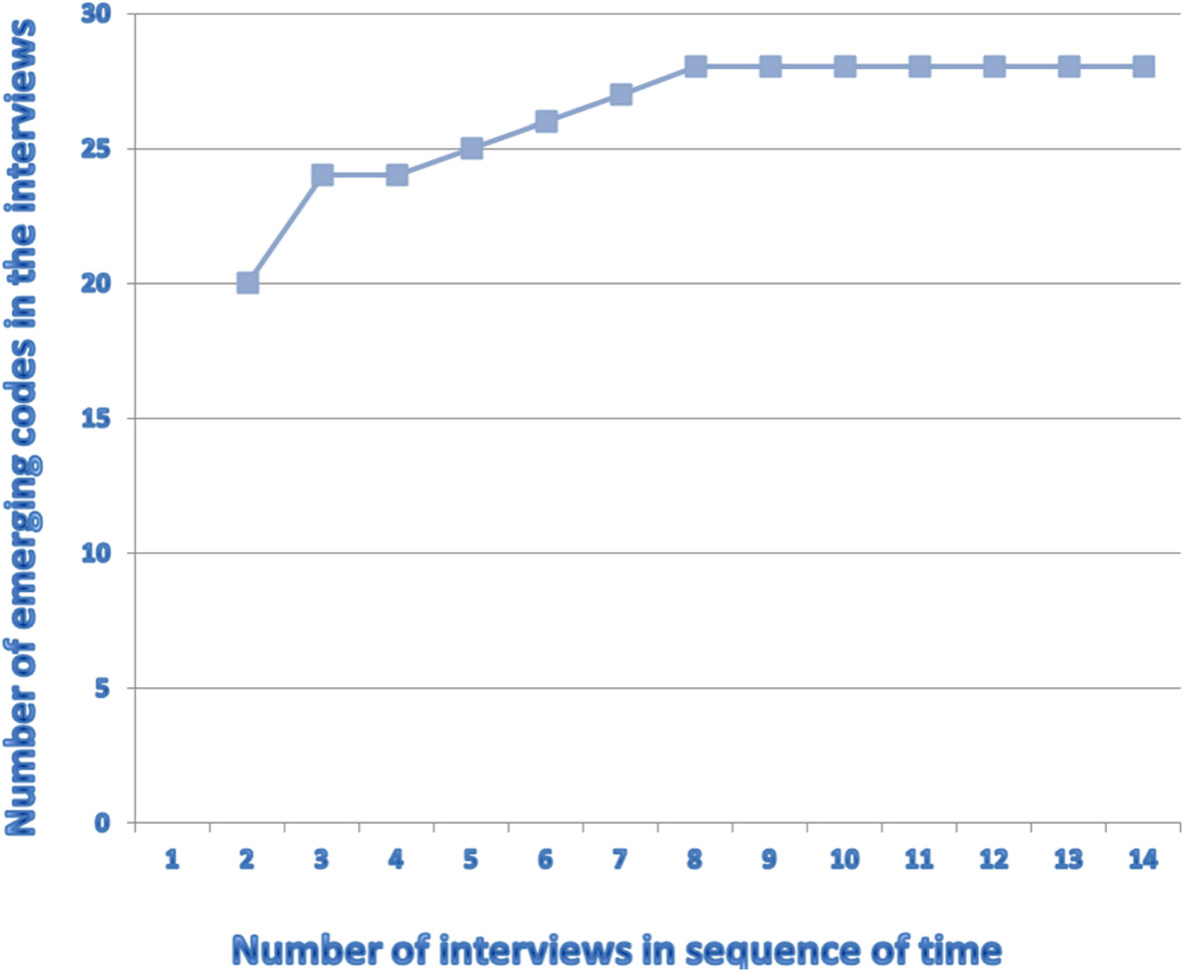
Saturation of codes in interviews

Three interviews were independently open coded by two researchers, following the conventional content analysis method [10]. Based on a comparison of the results, the Primary Researcher designed a preliminary codebook in cooperation with an Ethicist. Two researchers analysed all interviews with the help of this code book, using Atlas.ti 6.2.28^©^. Codes were checked by sample. Codes were refined through constant comparison. Consensus was reached on the attribution of the codes to the quotations. No new codes emerged in Interview 8, thus, saturation [11] was reached (Fig. 1). Finally, codes were concentrated in categories and combined to themes related to the research questions.

## Results

### Characteristics and typology of participants; interview themes

Table 1 gives an overview of the study participants. The 14 cases were divided into two groups: seven families informally knew the preferences of the deceased, the other seven families did not (see Table 2). In families with informal knowledge of donation preferences of the deceased, one family mentioned that their deceased relative had a contra-donation attitude. Another family explained that the deceased was only in favour of living donation to a well-known person. These two families refused consent for donation in line with the deceased’s preferences (Tables 1 and 2, Type A). The remaining five families with informal knowledge of donation preferences of the deceased mentioned that their deceased relative had a pro-donation attitude; one family initially gave consent for donation, but withdrew during the procedure; another family could not reach agreement and consequently refused consent for donation (Tables 1 and 2, Type B). Only one family completely adhered to the deceased’s preference and gave full consent for donation (Tables 1 and 2, Type C). Two families consented for donation after circulatory death (while donation after brain death was possible) (Tables 1 and 2, Type D). Of the families who did not know the deceased’s preference, only one family decided on consent for donation (Tables 1 and 2, Type E), the other six refused (Tables 1 and 2, Type F). For illustrative quotes, the participants’ type is denoted by the capitals A-F (Table 3).

**Table 1:** Eligible donors and relatives

Study code of eligible donor (N = 13; 14 interviews)	Sex/age	Days in hospital	Critical injury\illness	Preference on donation	Study code of participants (=Respondent) *N* = 24	Relation to (potential) donor	Sex/age	Type donation	Division of families	Type of decision^a^
P34	M39	5	hemorrhage	neg	R28	spouse	F 34	none	preference (of the eligible donor) known to families	A
					R29	friend	F 34			
P45	M26	5	Head injury (car accident)	neg	R33	spouse	F 21	none		A
					R34	mother in law	F 54			
					R35	father in law	M 52			
P32	M52	1	hemorrhage	pos	R25	spouse	F 50			
					R26	son	M 18	none		B
					R27	daughter - letter	F ?			
P49	F45	12	hemorrhage	pos	R41	sister	F 51	none		B
					R42	brother in law	M 51			
P05	M 43	16	hemorrhage	pos	R08	partner	F 52	DBD		C
P11	M 44	13	head injury (bike accident)	pos	R13	spouse	F 44	DCD		D
					R14	brother in law	M 49			
P38	M64	1	hemorrhage	pos	R30	spouse	F 58	DCD		D
					R31	daughter	F 28			
P01	M 54	8	hemorrhage	unk	R01	sister	F 53	DBD	preference (of the eligible donor) not known to families	E
P04	M 64	9	hemorrhage	unk	R07	daughter	F 31	none		F
P22	F 4	1	Oxygen deficiency	unk	R19	father	M 35	none		F
				unk	R20	mother	F 32	none		F
P23	M59	13	hemorrhage	unk	R21	sister	F55	none		F
					R22	sister	F55			
P31	F45	3	hemorrhage	unk	R23	mother	F72	none		F
					R24	sister	F48			
P42	M46	1	hemorrhage	unk	R32	spouse	F 47	none		F

*Abbreviations*: *neg* negative, *pos* positive, *unk* unknown, *DCD* donation after circulatory death; *DBD* donation after brain death

^a^Type of decision: see typology in Characteristics and typology of participants; interview themes section

**Table 2:** Division of participants

Number	Preferences eligible donor (un) known	Wishes or opinion on OD of eligible donor	Decision relatives	Manner of OD	Type of decision^a^
14 cases	Preferences eligible donor known for 7 families	2 contra OD	2 refusal	2 no OD	A
		5 pro OD	1 initial consent; later refusal 1 refusal OD	2 no OD	B
			3 consent OD	1 DBD	C
				2 DCD	D
	Preferences eligible donor not known for 7 families	7 not known	1 consent OD	1 DBD	E
			6 refusal OD	6 no OD	F

*OD* organ donation

*DCD* donation after circulatory death

*DBD* donation after brain death

^a^Type of decision: see typology in Characteristics and typology of participants; interview themes section

**Table 3:** Quotations divided in categories– with indication of type of participant

Q no	No resp	type^a^	
Request factors			
1	R24	F	R24. We cannot handle this right now. And maybe the way they put it, so soon after communicating that ‘there is no cure anymore’. Maybe that too… My mother had not yet recovered from…
2	R25	B	R25. That came right on top of it: “he does not have a codicil.” Well, all in the one sentence I think it was. That is why I say, I really feel bad about it. It is like; let us go to him for a moment first.
3	R33	A	R33. And then we got [the question] in the corridor: “what do you want?” And, really, no more information at all after that.
Behaviour of care professionals			
4	R23/R24	F	R23. It is just, I have a bad feeling about the intensivist that we were dealing with. I think she was a real cool and cold lady and she… R24. She was hardly humane. Just a medical…
5	R31/R30	D	R31. I think it also matters who delivers it [the death notice]. We just had… R30. A very kind doctor. R31. A very good one and she was really nice too. She told us in a gentle way, very nicely, but very clearly. Well, there was no doubt about it.
6	R27 (letter)	B	R27. That Monday was a very difficult day, and it is still awfully hard. The way we were treated at the hospital contributed to that. The fact that I was very afraid that Dad might still be able to hear something or be able to think, but could not move his body, was immediately cut short by the doctors and was considered rather ridiculous. …but it was my father lying there on that bed! We had to say goodbye to him in the end. Of course, I wanted him to have as little pain as possible, but because of the ambiguous, ‘cold’ and ‘ridiculing’ reaction I still wonder whether he really did have as little pain as possible.
7	R25	B	R25. Well, perhaps then, we would have made a few different choices. Because the doctor made his announcement and then ‘*whoosh’*, he was gone again. ‘You can take your time thinking about it, I will be with you in a minute.’ Then another doctor appeared, and yet another. You hardly knew who they all were.
8	R41	B	R41. So then in the end we said….well…They discussed it with their two children and her ex-husband and I discussed it with the doctors about whether we should give consent or not. They did try to persuade us a little bit, saying that it was a way of helping other people and all that, but actually the children and the husband were really very much against it, so that is what I told them.
9	R25	B	R25. [They said]: “He does not have a codicil,… but we can use all the parts of his body.” At one point I said, “Well, not his lungs surely, since they must be completely black from smoking,”. To which, they replied: “Well, that is rather silly madam, because we can even use those.” So I was thinking “Snatch it all away!” That was really how it felt like then.
10	R34	A	I think that at that moment she said to me, we can make a young man of 20 very happy with his heart; I think that she perhaps would have listened…
11	R08	C	R08. Well, then his parents arrived. They were here for three days, and then his brothers arrived. I asked the hospital whether a team of doctors could be present when I arrived with the parents. I wanted the parents to be well- informed, so that they would finally understand that things were not going well. Because his father said: “Look, my son will pull through. Next year you will be with us on our holidays.” And I thought: “That is not going to happen.” Yeah, well, I suppose losing a child is worse. Because a child leaves your heart, and a husband leaves your side, you know. They did not want to understand.
12	R20	F	R20. Then everybody was very angry: ‘This really can’t be happening!’ We had just arrived at the hospital. They said: “Three days, and then we‘ll see”. And now, all of a sudden, the treatment stopped. She is going to die. She is just going to die. I am thinking: “This cannot be happening. My little girl cannot die!”
13	R25	B	R25. We had to ask [the doctors] ourselves if we could take a look at the scan. Because you just cannot believe it. I thought: “He is lying there. It is just like he is lying asleep on the couch at home. He looks completely normal.” Just let me see that scan, we only wanted something…
14	R41/R42	B	R41. Yeah, at that moment you are overwhelmed with grief, gosh, she really has died. My parents had not yet realised that she had died. I told them then… that this means… {Interviewer: in fact, she is already dead} R41. That she is actually already dead. “No,” my father said, “She is still breathing.” “But Dad, [she is] not [breathing] herself anymore. “Yeah, yeah, yeah” [acts like her father, hesitant, unsure] {Interviewer: people do not get that; you do not get that…} R41. [acting like father] “And she is still warm. And she is still warm and still breathing. No, she is not dead yet,” I said: “But she certainly is brain-dead.” “No, her heart is still beating, she is not dead yet.” So… R 42. [He is] 77 years old. R41. 77 years old, so yeah, he did not really comprehend it.
Prior knowledge and opinions			
15	R08	C	R08. That is what we thought. Just imagine that you have something like that [organ failure] yourself and you have been waiting several years for it [an organ]. Can you imagine how happy you would be to receive a kidney, for example? (…) More arguments? Yes. He also had a friend who had been on dialysis for several years, waiting for a kidney.
16	R30	D	R30. That was really important for me: that none of us would have trouble with it afterwards. Because if one of us would have said: “Mom, I have a problem with that”, then I would not have done it. No, because I think that is really important, since the four of us have to live with it. Yeah, that was really important to me.
17	R31	D	R31. He [deceased] always said…: “Dead is dead. When I am dead they can have all of my body. But I will leave you to decide on that, since you are the ones who will have to deal with all the hassle.”
Relatives’ decision making			
18	R20	F	R20. And the moment as well: you are really in shock. First you had a healthy child, and your baby was always around, and she lived and was hardly ever ill. And all at once you are in a situation where everything is so difficult to comprehend, it is in fact incomprehensible. And then you have to make such a choice too. Then that [choice] is easily made actually, since you quickly say: “No, we will not do it.” Then you do not have to mull over it any more. You can go on with what you were doing: being with her [daughter].
19	R41/42	B	R41. Her ex-husband had fewer problems with it, but the children did not want it [donation] in the end. So then I said’Well, let them decide. We will not do it.’ R42. Though we do think it is a pity. R41. Though we do think it is too bad.
20	R31	D	R31. I wanted to be sure. I wanted to see him die. I just could not believe what was happening, and I think that if he had gone to surgery alive, well… Yeah, well, alive for us I mean. I think that would not have been…
Evaluation of decision (process)			
21	R21	F	R21. If we had been prepared a bit, it would have been totally different. It would have been a completely different story. You start thinking about it and talking about it with each other. So your circle of people gets bigger when you have to make a decision.
22	R32	F	R32. But if that person [doctor] had said… Well, you know, when the doctors say like… well, it is kind of a cold process. You donate a heart and then… He goes cold, uh, warm into the OR and comes back cold. That could also be told in a different way. In a way with more room for alleviating circumstances. (…) Such nuances do make a difference, I think. Maybe I would have said then, well, take his kidneys, yeah, uh…
23	R41	B	R41. Yes, and then I am right in the middle of it [the family who had to make a decision]. I managed it all right. I organised everything and all, but yes [the family was flabbergasted]. But I did ask for help at one point, and I did not get it.

^a^for explanation of type –see Characteristics and typology of participants; interview themes section and Table 2

**Fig. 2:**
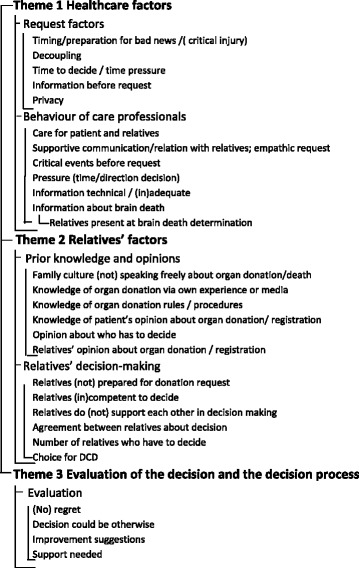
Code tree

Twenty-eight codes were identified (marked in italics in this article), concentrated into five categories, resulting in three themes (Fig. 2; Table 4). The first theme concerned the healthcare system: request factors and requestor factors. The second theme comprised items related to the relatives: prior knowledge or opinion about organ donation and their decision-making. The third theme related to all factors concerning the evaluation of the decision and the decision process. Representative quotes of identified codes are presented in Table 3 and are referred to in the manuscript as ‘Qn’.

**Table 4:** Code book

Theme	Category	Code interview	Definition	Density^a^
Health care factors	Request factors	Surprised by bad news/timing of the request	How the timing of the donation request influenced the decision, ‘bad timing’, whether the participant was prepared for the request or surprised	14
		Decoupling	Whether or not communicating the death of the patient and making the donation request took place in a single conversation or not, and how this affected the participant	11
		Time to decide/time pressure	Enough time for the relatives to discuss donation or the feeling of time pressure	12
		Information on patient before donation request	Whether the participant felt he was well-informed about the treatment and the treatment decisions that were made previous to the donation request. This affected the preparedness for the request and the trust the participant had in the care professional	6
		Privacy	Where the request was made: in a separate room/in the hallway/next to the bed/etc. and how the participant felt about this	5
	Behaviour of care professionals	Care for patient and relatives	Remarks on quality of care for patient and relatives of patient	13
		(Supportive) relationship and communication with relatives; empathic request	Participant stated how he experienced the relationship with the care professionals during the stay at the hospital and whether this influenced the decision-making process; was the request done in an empathic way.	12
		Critical events before request	Things that went wrong which caused stress or distrust for the family before the donation request was made: communication errors, transmission to other hospital/ward, messages of hope from healthcare professionals, long stay in the hospital before brain death	12
		Pressure (time/direction decision)	Participant mentioned pressure by professionals to make a (fast) decision, or other factors that put pressure on the decision	6
		Information (in) adequate/technical	The amount, the dosage and the quality of information on organ donation provided by the physician and whether the participant understood it well enough to make a good decision on organ donation.	12
		Information about brain death	Whether the participant understood the concept ‘brain death’, how this concept was explained and whether he and other relatives understood that the patient was dead.	13
		Relatives present at brain death determination	Participant and/or other relatives were (not) allowed to be present when brain death was determined. This influenced the acceptance and understanding of the death of the patient.	4
Relatives’ factors	Prior knowledge and opinions	Family culture (not) speaking freely about organ donation/death	The family members (did not) talk(ed) about organ donation, death, funeral, last wishes in daily life, before admission to the hospital. Quotes on how accustomed family members were to talk about difficult subjects. E.g. ‘we do not talk much at home’ or ‘we always had discussions on everything during dinner. ‘	14
		Knowledge of organ donation via (own) experience or media	Participant knew about organ donation and/or the procedure before arriving at the hospital because a) he knew people who donated, received an organ or are on the transplant list; b) he heard of organ donation in the media or through education or information campaigns.	9
		Knowledge of organ donation rules/procedures	Participant knew rules concerning organ donation (for instance: organ distribution system, no contact with receptor, no influence on destination organs etc.) and procedures (e.g. brain death notification, length of procedure etc.)	4
		Knowledge of patients’ opinion about organ donation/registration	The participant (did not) know/knew the deceased’s preference and/or knew why/that the deceased had not registered himself as donor in the donor register.	12
		Opinion about who has to decide	Opinion of relatives of deceased about who had the decisive say about the organs: the deceased or the relatives	5
		Relatives’ opinion about organ donation/registration	The participant himself was (not) in favour of organ donation (with motivation) and was (not) registered in the National Donor Register. The other relatives were (not) in favour of organ donation and were (not) registered.	14
	Relatives’ decision making	Relatives (not) prepared for donation request	Participant mentioned that they saw the donation request coming, or that they were totally surprised by it.	14
		Relatives (in)competent to decide	Participant was (not) able to think clearly/was very emotional/did (not) get the information	9
		Relatives (do not) support each other in decision making	Participants are the legally appointed representatives of the deceased. Other family members also play a part in the decision making process, in favour of or against the opinion of the participant. Participants can also have a caretaking role towards relatives, which created an extra burden.	12
		Agreement between relatives about decision	How relatives reached agreement on organ donation or not.	13
		Number of relatives who have to decide	Number and composition of relatives present at donation request conversation and/or involved afterwards in the decision-making process on organ donation	14
		Option of DCD instead of DBD	Participants made a choice for DCD instead of DBD because they wanted to be present at cardiac arrest or did not want to wait long(er) for a DBD-procedure	14
Evaluation decision (process)	Evaluation of the decision and the decision process	(No) regret	Participant mentioned that he did (not) regret the decision and/or that he was proud of the decision and the way it was made. The decision did justice to the preference of the deceased.	14
		Decision could be otherwise	Participant stated that the decision to donate could have been otherwise (without regretting the decision made).	9
		Improvement suggestions	Participant mentioned improvements: they needed more information, more time to deliberate with others, more (empathic) support from care professionals, they did not know to whom they could address questions etc.	13
		Need for support	Participant mentioned that he would (not) have wanted counselling himself, or that he asked for support. Participant could imagine that other people might need counselling, or that he might have needed counselling if the situation had been different.	12

^a^Number of interviews with this code

### Healthcare system factors

#### Request factors

Nearly all families mentioned a lack of time to recover from the news of the death of their relative and their *surprise by the request for consent to donation*. Those who refused consent for donation mentioned that a separation in time (‘decoupling’), between the notification of death and the donation request was desirable (Q1). None of our participants experienced the *decoupling* in the communication with doctors. Families with informal knowledge of donation preferences of the deceased and a positive donation attitude considered this an omission (Q2). Other factors mentioned as contributing to refusal were: needing more *time to decide* whilst feeling *pressured* to make a quick decision; inadequate or no *information preceding the donation request*; and a lack of *privacy* (Q3). In contrast, appropriate *timing* of the request was mentioned as a factor leading to consent to donation.

#### Factors related to behaviour of care professionals

Families were generally content with the *care for the patient*. They felt that *care for the relatives* could, however, be improved. Poor care for the relatives even compelled some families to refuse consent for donation: they had to wait a long time for the physician, he/she was rather aloof in his/her attitude (Q4), or there was no support for grieving parents. An empathic and *good relationship* with the staff made the whole experience less stressful for relatives, especially for those who consented to donation (Q5). If the request was experienced in a less empathic way, this factor was given as an extra reason for refusal of consent for donation. A poor relationship with the caregiver, sometimes induced by a *critical event* in this relationship (Q6), a less *supportive attitude of the requestor* and too many changes in caregivers (Q7), were related to refusal of consent for donation.

Families with informal knowledge of donation preferences of the deceased experienced more *pressure* to donate organs (Q8). When the requestor had a utilitarian approach (Q9) or emphasised the interest of persons on the waiting list, some relatives refused consent for donation, or did not want to discuss donation at all. However, other participants, who refused consent for donation, would have appreciated more emphasis on the benefits of donation (Q10).

Giving *adequate information* was seen as an important part of good care. Participants mentioned sometimes that information did not sink in, due to emotions (Q11), or because it was too technical for them*.* Some were still upset because they understood the received information as contradictory (Q12). Relatives who received adequate information were better able to understand the seriousness of the situation; they realised that their relative was going to die and understood the concept of brain death.

Participants deemed clear *information about brain death* as especially important. All families who consented to donation reported that they understood the meaning of brain death, whilst some of the families who refused consent for donation required more proof that their brain dead relative was ‘really dead’ (Q13). Other refusers rejected the concept of brain death entirely. (Q14).

### Factors related to relatives

#### Prior knowledge and opinions

The outcome of the donation request was influenced by prior knowledge and opinions of the relatives. Families who did not know the deceased’s preference or who were not *accustomed to speak freely* about death and funeral wishes, were often not aware of each other’s donation preferences and often rejected donation after brain death. Families who knew the preferences of the deceased, on the other hand, usually talked openly about these themes. Donation *campaigns and documentaries in the media* were mentioned as the immediate reason for a conversation about organ donation in the family, or the decision to register as donor. Another source of prior knowledge was *acquaintance with someone* on the transplantation list. This was a reason for families to donate (Q15). Some participants complained about lack of public information on *donation rules and procedures.* Their prior knowledge on this topic was very limited. Once relatives consented to donation, these rules and procedures had to be explained; especially the length of the procedure was an unexpected disappointment. In one case, relatives refused consent for donation when they learned that the recipient would remain anonymous, although their deceased relative was in favour of donation.

Opinions of the deceased and his/her relatives regarding donation can be different, and so hamper consent: even when families know the explicit *preference of the deceased to donate*, they did not always follow it automatically. These families felt that *their opinion on donation was more important* than the (last) will of the potential organ donor, because they would have to live with the decision (Q16). Sometimes, the eligible donor had foreseen this situation and decided to allow his/her relatives the freedom to decide, irrespective of his/her preference to donate (Q17). Some participants refused to register themselves for the same reason*.* Moreover, even when the *relatives were registered donors* themselves, they did not automatically choose for consent to donate as a surrogate decision.

Thus, lack of prior knowledge and conflicting opinions contributed to refusal to consent to donation.

#### The decision-making process by relatives

The decision-making process was constrained when relatives *were taken by surprise* by the request, when they were in shock and felt *incompetent* (Q18) to make a decision. Relatives inclined to refuse consent for donation when they received less *support from each other*: some participants had to take care of children or parents*,* support other relatives in their mourning, or deal with disagreement in their families*.* These participants felt unsupported in their decision: other relatives refused consent for donation; their deceased relative left them without advance directive; family conflicts resurfaced. The donation request was seldom done to the legal representative(s) alone, but mostly in the circle of more relatives. Agreement between relatives was mentioned as necessary condition by most participants who had jurisdiction. Thus, *disagreement between relatives* automatically led to refusal (Q16). Two participants, who knew the deceased would have wanted donation, regretted that they had refused consent to donation because of disagreement. (Q19). The *number* of people engaged in the donation decision did not seem to influence the decision either way.

Although some families of donation-minded patients felt positive towards donation, they decided not to follow the preference of the potential organ donor and chose donation after circulatory death instead of donation after brain death, whereas full donation was possible (Type D). They did not want to wait long(er) or they wanted to be present at the moment of visible death (cardiac arrest) (Q20). When relatives refused consent for donation the *option of donation after circulatory death instead of donation after brain death* was not offered to the participants, nor deliberated by them, not even when it was clear that the refusal arose from the long procedure or the wish to be present at death.

### Evaluation of the decision and the decision process

Although there was hardly any *regret* about refusal, participants that refused consent for donation remained ambivalent during decision-making and said that their *decision could have been different,* if the above-mentioned relevant factors had been different (Q7; Q21; Q22). These factors were also mentioned by some others, when asked for *suggestions for improvement.* Participants confirmed the importance of more time between the death notice and the consent for donation request (decoupling). They stated that they needed information to be provided on time and in proportion to their capacity to understand, since they could not understand it all the first time. They suggested putting leaflets about brain death and organ donation in the waiting room, so that they could be prepared for the possible request and reread the information afterwards. Also, they thought it would help them to understand brain death if they were allowed to attend brain death determination or when brain scans were shown and explained.

Consenting families often evaluated the donation procedure negatively, because they had to wait a long time for brain death to occur and/or be established. Participants of all groups pleaded for more information about donation with special attention to the length of the procedure, because none of them were aware of this. In retrospect, some participants would have appreciated extra time or *support from a professional caregiver*, since there was disagreement or little support from other relatives (Q23).

### Metaphoric summary of the findings

**Fig. 3:**
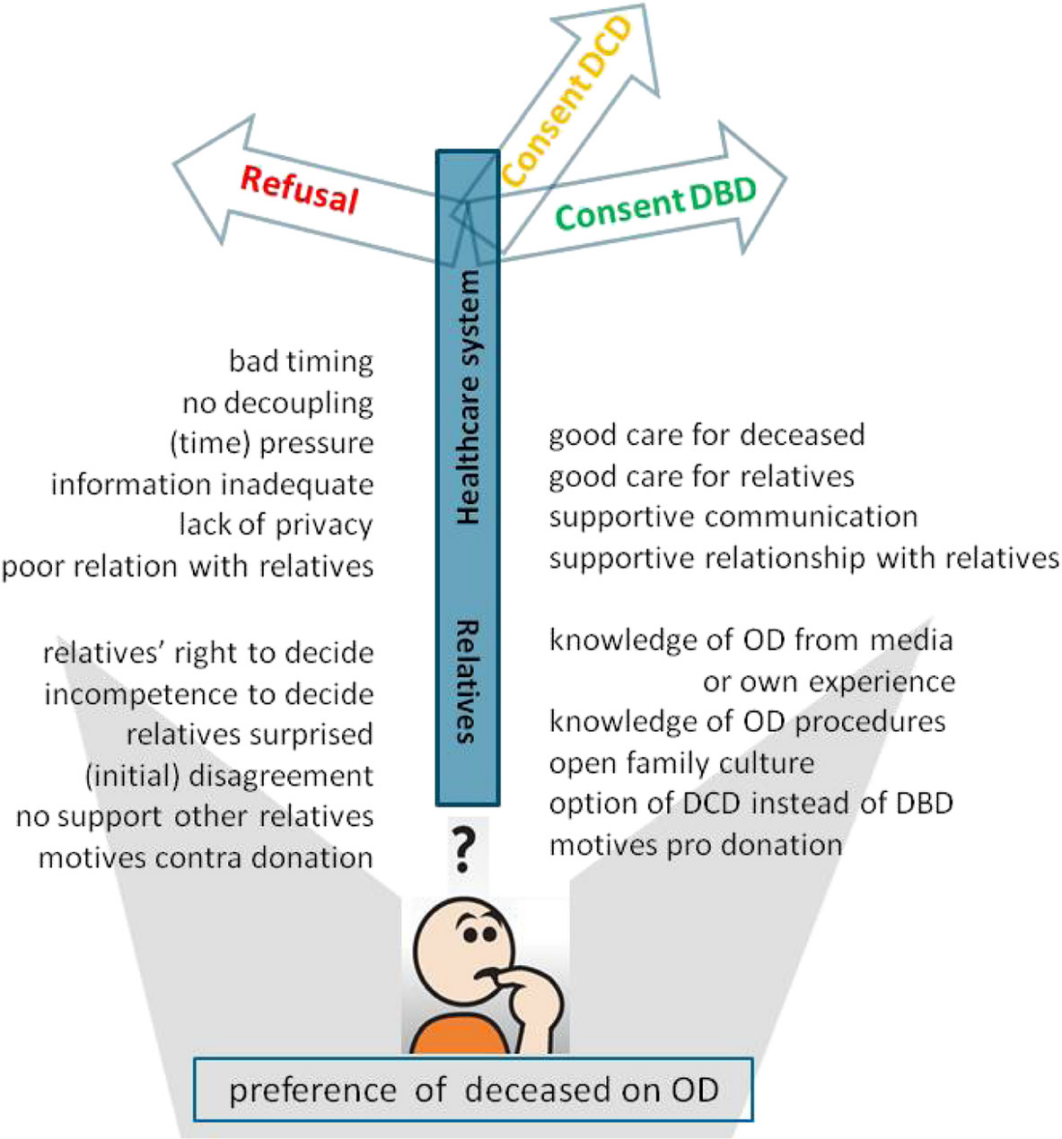
General model showing factors that influence the decision process for organ donation of relatives. General model. A participant who has to choose between (left) donation refusal or (right) consent to DBD (or DCD, when they had reasons to refuse DBD). Beneath the signpost, all factors are listed that could contribute to consent or refusal to consent to donation in absence of the registered preference of the deceased: top the healthcare-related factors, bottom the relative-related factors. Abbreviations: DCD = donation after circulatory death; DBD = donation after brain death; OD = organ donation

Although this study has an exploratory character only, it can be used to develop a suggested model that combines all remarks as a kind of summary. It uses a metaphor and picture of the participants on the crossroad of their decision process (Fig. 3).

**Fig. 4:**
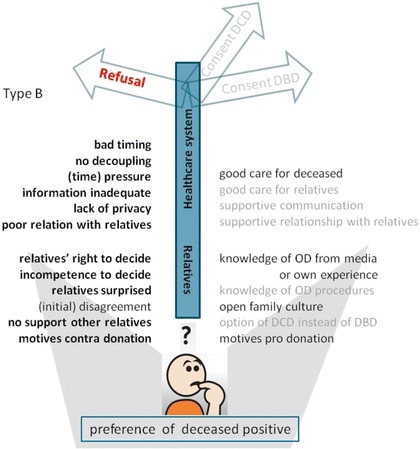
Model of factors influencing the decision towards refusal of consent for donation, although the deceased was in favour of organ donation. Model for families who did not comply with the deceased’s preferences (type B, see Table 2). Important factors are denoted in bold script, absent factors in faded script. Abbreviations: DCD = donation after circulatory death; DBD = donation after brain death; OD = organ donation

**Fig. 5:**
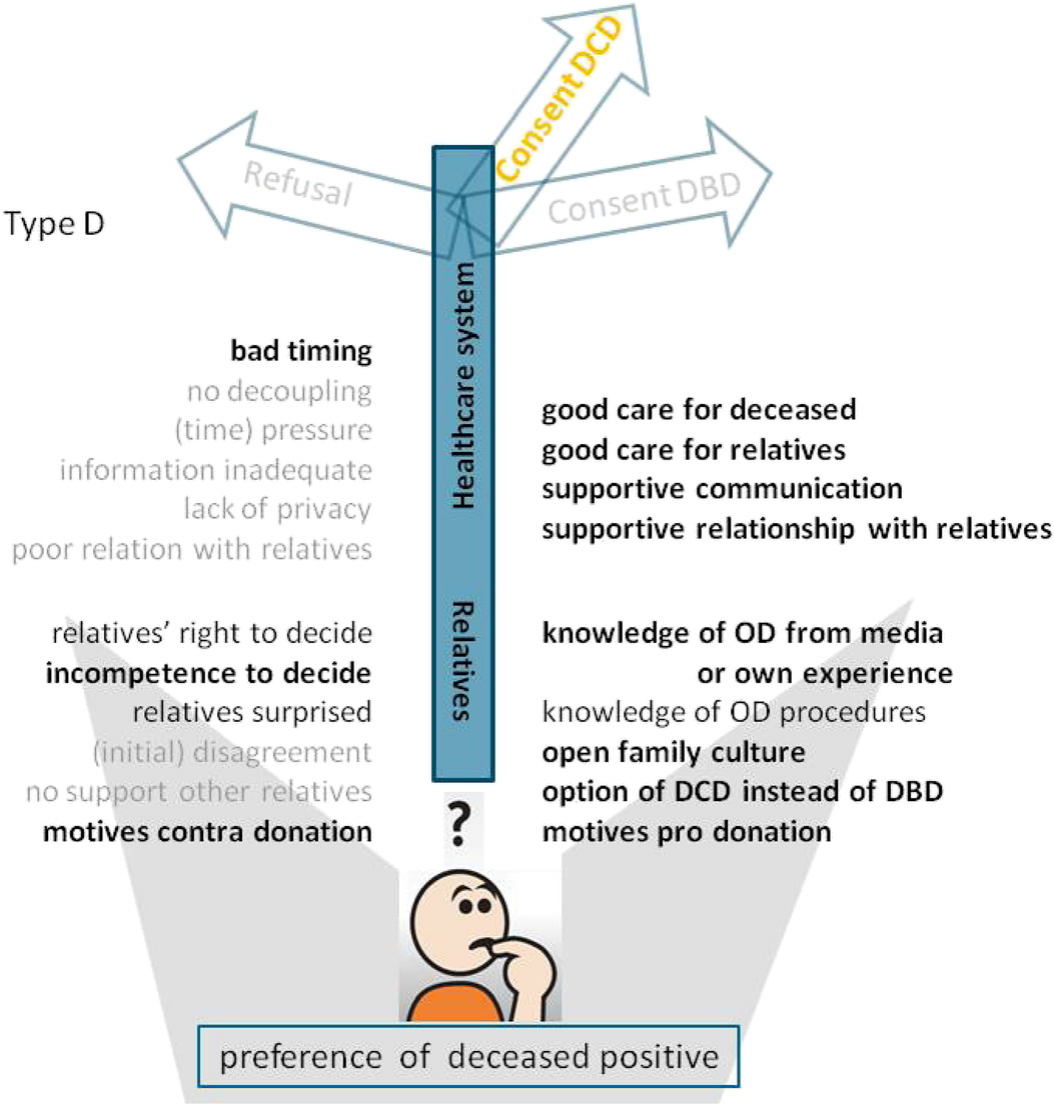
Model of factors that influence the decision towards consent for DCD, whilst DBD was possible, although the deceased was in favour of organ donation. Model for families who did not completely comply with the deceased’s preferences (type D, see Table 2). Important factors are denoted in bold script, absent factors in faded script. Abbreviations: DCD = donation after circulatory death; DBD = donation after brain death; OD = organ donation

**Fig. 6:**
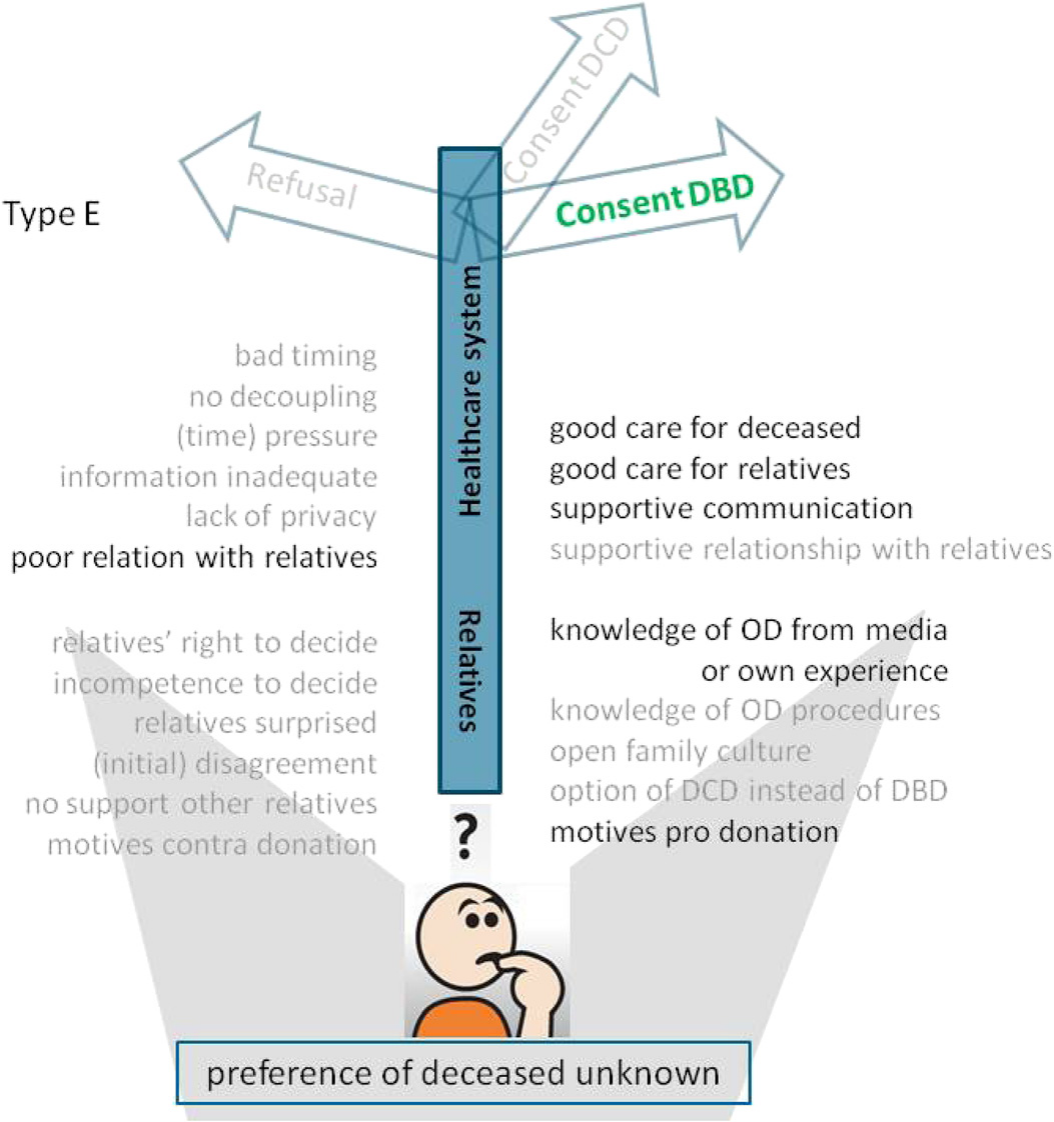
Model of factors that influence the decision towards consent for DBD, whilst the preference of the deceased on organ donation was unknown. Model for families who did not know the deceased’s preferences and gave consent for organ donation (type E, see Table 2). Important factors are denoted in bold script, absent factors in faded script. Abbreviations: DCD = donation after circulatory death; DBD = donation after brain death; OD = organ donation

**Fig. 7:**
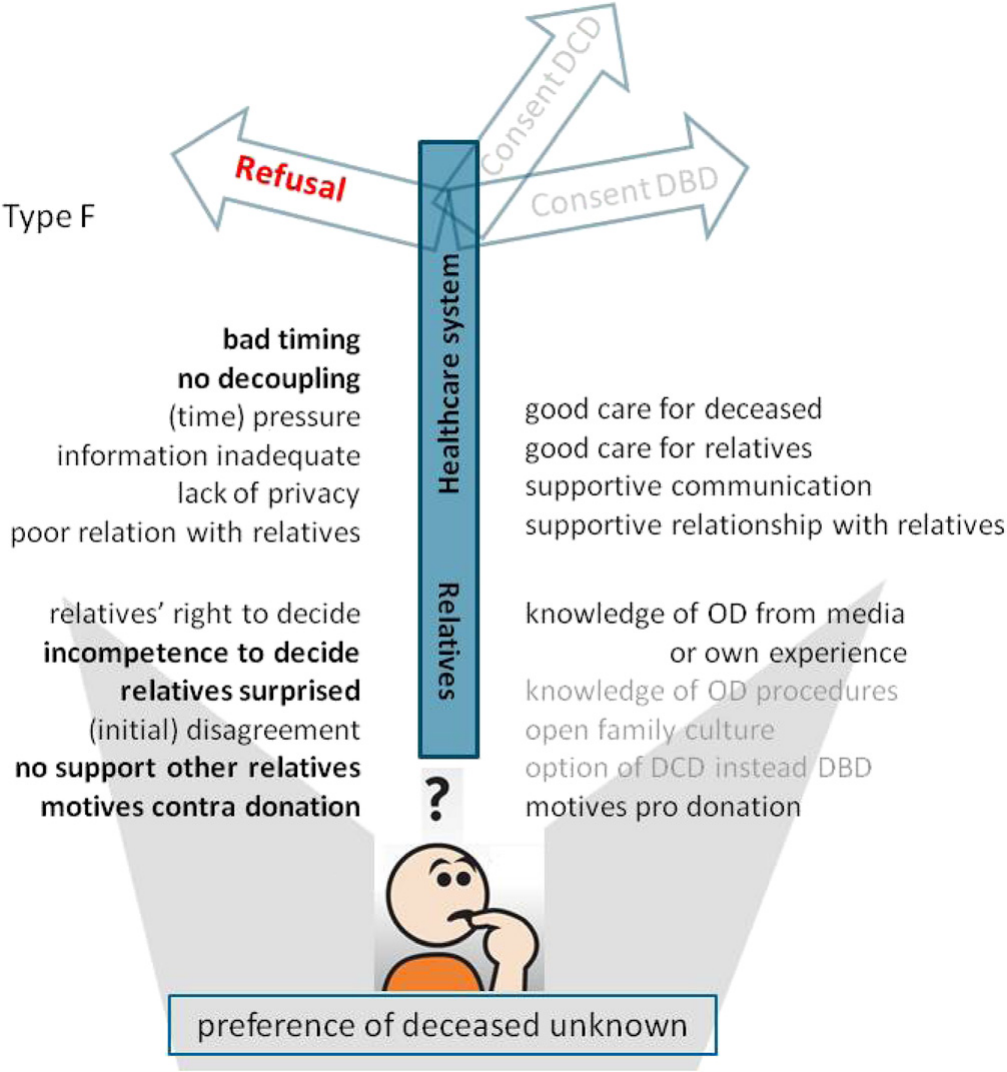
Model of factors that influence the decision towards refusal of consent for donation, whilst the preference of the deceased on organ donation was unknown. Model for families who not know the deceased’s preference and refused consent for donation (type F, see Table 2). Important factors are denoted in bold script, absent factors in faded script. Abbreviations: DCD = donation after circulatory death; DBD = donation after brain death; OD = organ donation

In additive figures, this model was applied for respondent types who made a decision that deviated from the deceased’s preference (Figs. 4 and 5) and respondents who did not know the preference of the eligible donor (Figs. 6 and 7). Respondents who adhered to the deceased’s preference are not shown. Differences in starting situations gave a different weight to the contributing factors. The weight respondents’ gave to these factors is emphasised with boldness for important factors, and fading for absent factors.

## Discussion

In this study, a model of the decision-making by relatives of unregistered eligible organ donors is presented. The main interest is the decision (consent to or refusal to consent to organ donation) combined with stability of satisfaction about the decision. The model differs from the thematic analysis of Ralph et al. [12], because its main focus is on situational factors. In a previous publication [7], the weighing of values in the decision process was explored, and the conclusion was that ambivalence about the decision continues to exist. In those circumstances, situational factors were pivotal. In this model, several factors are discerned; firstly, those located in the healthcare system and secondly, factors that are connected to the relatives themselves.

With respect to factors located in the healthcare system, participants stated that they needed more time to recover from the notification of death before considering the donation request. Creation of a time span between the notification of death and the request for donation (decoupling) is advised in literature [3, 13–16], but is not standard practice in the Netherlands [17]. Remarkably, the participants in this study did not recall having this time span, though decoupling is common practice in the participating hospitals according to the physicians. The difference can be explained by the fact that the study involved grieving participants, who sometimes recalled only vague details of the elapsed time [18]. Therefore, more research on the actual time between conversations and how this time span is perceived differently by healthcare professionals and relatives is required. Participants confirmed the frequently-mentioned importance of time, timing and adequate information concerning the request [9, 19]. They confirmed that it is important for requestors to take time to explain the donation procedure and to give relatives time to make a decision [14, 15, 20–22]. Perhaps relatives’ need even more time, both for decoupling and after the donation request than the medical procedure of organ procurement allows. Besides lack of time, the participants stated that healthcare professionals were not always aware that they did not understand the concept of brain death [16, 23]. Some relatives were emotionally incapable of receiving this technical information, some needed proof their family member was dead, others did not believe in the concept brain-death. Not understanding or accepting the concept of brain death and the requirements for a donation after brain death procedure led to a decline in consent for donation in this study. Participants also refused consent for donation when they felt that the potential organ donor was seen as an object instead of a subject; a utilitarian or organ-focused approach harmed relatives’ relationship with healthcare professionals, as endorsed by other studies [24, 25]. Physicians might not recognise this approach as a source of conflict that causes unnecessary misunderstandings [26]. For the participants in this study, the length of the procedure was an important obstacle, which was also shown by Thomas et al. [15]. This stumbling block could be overcome by offering donation after circulatory death as a possibility for those who would otherwise completely refuse consent for donation. donation after circulatory death could also be an option for relatives who wish to witness the heart beat cessation or who do not wish to donate the heart.

Regarding relative-related factors, healthcare professionals should be aware that participants tend to refuse consent for donation when they feel a lack of support from their relatives in combination with a lack of professional support. Extra professional support leads to more consent and more satisfaction about the decision [27]. Most of the families who did not know the deceased’s preference would have appreciated better and more information and supportive care, as was suggested in the reviews of West et al. and Walker et al [16, 19]. Good supportive care for all relatives before the request increases the possibility of consent.

A striking fact concerning this second aspect, was that the preference of the eligible, but unregistered donor was not always the decisive factor, in contrast with findings in literature [1, 2, 4, 28]. That preference was known in half of the cases studied. Remarkably, in the sample, knowing the positive, but not registered preferences of the deceased did not automatically lead to consent. Posthumous autonomy in these cases was not seen as granted [29] by the relatives who told they had to live with the consequences. That option has been ethically-defended by Wilkinson [29, 30], but contradicted by Bramstedt [31]. Some families considered the fact that the eligible donor was not registered to mean that they could decide for themselves. Of great importance in surrogate decision-making is whether the surrogates are used to openly speaking about death themes (burial, donation, end-of-life decisions) [32, 33]. Familiarity with death themes facilitates the group discussion. Although the Dutch legal system attributes jurisdiction to (a) specific person(s), agreement of all involved relatives was necessary to decide in favour of the request. All but one of the families who did not know the deceased’s preference refused consent for donation. Surprisingly, the relatives did not accentuate more negative factors than consenting families; they only mentioned less positive factors, such as supportive communication, relationships and care. Contributing to more positive factors could therefore be a better way of preventing refusal and possible regret about this decision, rather than focusing on negative aspects.

### Strength and limitations of this research

In this research, a broad spectrum of opinions was discovered within a small sample of participants, who were vulnerable, because of their grief, and often refuse to participate in research [34]. A considered and complete overview was compiled by reaching saturation of topics (codes). The decision process of relatives of unregistered eligible donors who knew the deceased’s donation preferences was compared to those who did not know these wishes. The majority of the relatives refused consent for donation. This makes the sample unique, but could also give a negatively-biased image of the donation practice. Studies, in which the majority of the respondents consented, might give a more positive view, since donors are in general more content with the donation process than those who refuse [3]. However, the study provides a unique overview of factors attributed to the decision, presented in a model. This model needs further research, because the study was explorative. This research underscores the desirability of support for relatives, before and during the request period. It nuances the opinion that the deceased’s’ preference is key in the relatives’ decision, because families of unregistered eligible donors often make their own choice.

## Conclusion

The decision not to consent to donation is attributed to contextual factors bad timing of the request; feeling overwhelmed; insufficient support from other relatives or health care professionals; little knowledge on organ donation (especially on the length of the procedure). These factors are more heavily weighted when the preference of the deceased is unknown. Even when it is informally known that the deceased favoured organ donation, relatives may ignore that wish in the absence of official registration. Healthcare professionals could provide better support for the relatives prior to the donation request, address their informational needs and adapt their message appropriately, especially when relatives are not familiar with talking about death themes. The study findings show that more satisfaction regarding the decision can be expected if relatives experience decoupling, and consecutively more consent when the possibility of donation after circulatory death is offered to those families who otherwise would have refused brain death donation.

## Additional files

Additional file 1: Information about organ donation and transplantation in the Netherlands. (DOCX 15 kb) Additional file 2: Topics for the interviews. (DOCX 14 kb)
