# Supplementary material for: Request for organ donation without donor registration: a qualitative study of the perspectives of bereaved relatives
Source: BMC Med Ethics. 2016 Jul 11;17:38. doi: 10.1186/s12910-016-0120-6 (PMC4940748; doi:10.1186/s12910-016-0120-6)
Supplement: Additional file 1: — Information about organ donation and transplantation in the Netherlands. (DOCX 15 kb) [file 12910_2016_120_MOESM1_ESM.docx]

| Additional file 1 – Information about organ donation and transplantation in the Netherlands | |
| --- | --- |
| The Netherlands: 17 million inhabitants^1^  Number of effectuated organ donors (average^2^) total 232^3^  of these: 123 DBD  109 DCD | |
| Donors per year per one million (average^2^): 14^3^ | |
| Law on Organ Donation since 24^th^ May 1996, revised 23^rd^ June 2006  Opt-in system (no presumed consent) with a plea for Active Donor Registration^4^ | |
| National Donor Register (NDR) since 1998  Donor registration in the register or by codicil is legally binding  Participation in the NDR^5^ : 5.8 million inhabitants (44% of all adults; 34% of total population)  choice in the NDR: consent for donation 61%  no consent for donation 27%  relatives decide 12%  In absence of registration in the NDR the jurisdiction in the Netherlands to decide on the request on organ donation is attributed to 1) the spouse, 2) children 3) the parents. If the wish of the deceased is not administrated in the NDR, the legal representative is not obliged to follow the wish of the deceased. | |
| Consulting NDR (average^2^) for organs and tissues^3^:  Total: 8,487  Not successful (patient not in register 4,845 (57%)  Successful (patient in register) 3,642 (43%) | Consulting NDR (average^2^) only for organs^3^:  Total: 611  Not successful 322 (53%)  Successful 289 (47%)  of these:  141 consented to donation (23%)  99 objected to donation (16%)  49 relatives had to decide (8%) |
| Organ donation activities (average^2^):  Participating donation hospitals: 84^3^  – 17 of them (including the eight transplant centres) have 75% of all organ donations  Transplant centers: eight University Medical Centres and affiliated children hospitals  Eligible organ donors: 709  Register consulted: 611  Donation obstructed by relatives:  when eligible donor had consented to donation via NDR 6%  when eligible donor was not registered or if register was not consulted: 67% | |
| ^1^ numbers from Central Office of Statistics in the Netherlands (CBS) (<http://www.cbs.nl/nl-NL/menu/cijfers/default.htm>)  ^2^ average of the last five years (2009-2013)  ^3^ numbers from Dutch Transplant Foundation ([www.transplantatiestichting.nl](http://www.transplantatiestichting.nl))  ^4^ source: Kidney Patients Association NL (nierpatiënten vereniging) <http://www.nvn.nl/zorg/wet-op-de-orgaandonatie>  ^5^ National Donor Register (NDR) <http://www.donorregister.nl> | |
