# Supplementary material for: Request for organ donation without donor registration: a qualitative study of the perspectives of bereaved relatives
Source: BMC Med Ethics. 2016 Jul 11;17:38. doi: 10.1186/s12910-016-0120-6 (PMC4940748; doi:10.1186/s12910-016-0120-6)
Supplement: Additional file 2: — Topics for the interviews. (DOCX 14 kb) [file 12910_2016_120_MOESM2_ESM.docx]

| **Additional file2: Topics for the interviews** | | |
| --- | --- | --- |
| nr | Interview topics | Conceptual background |
|  | Introduction: the process before the request for consent for donation; experiences of the proxies in the hospital. |  |
| 1 | Considerations to decide for donation on behalf of the potential donor. | Integrity,  non-maleficence. |
| 2 | The wishes of the potential donor concerning donation; the Dutch National Donor Register. | Patient autonomy,  self-determination. |
| 3 | The wishes and opinions of the participant concerning donation (by themselves). | Beneficence, justice,  easy rescue,  gift or sacrifice,  solidarity, altruism. |
| 4 | Need for coaching or (moral) counselling during the decision-making process; wishes concerning the profile of the counsellor. | Vulnerability,  crisis,  moral distress. |
| 5 | Review of the decision: peace of mind. | Dignity,  respecting the deceased,  pride in the decision. |
|  | Additional comments and evaluation of the interview. |  |
